# LAGGED ASSOCIATIONS BETWEEN DAILY SYMPTOMS OF OSTEOARTHRITIS AND PHYSICAL ACTIVITY LEVEL

**DOI:** 10.1093/geroni/igz038.732

**Published:** 2019-11-08

**Authors:** Dylan M Smith, Patricia A Parmelee

**Affiliations:** 1 Stony Brook University, Stony Brook, New York, United States; 2 The University of Alabama, Tuscaloosa, Alabama, United States

## Abstract

Fatigue is commonly reported by persons with lower limb osteoarthritis (OA) and uniquely predicts worse functioning and decreased activity. The current research used a combination of wrist and waist accelerometry along with experience sampling methodology (ESM) in a diverse sample to examine the relationship between reports of fatigue and pain and subsequent physical activity among older adults with knee OA. Three hundred twenty-one participants with physician-diagnosed knee OA completed a baseline interview and mobility testing followed by a one-week observation period using accelerometry at both wrist and waist. During this week, participants also completed an ESM protocol assessing fatigue and pain 4 times daily. Multilevel models examined lagged within-subjects patterns of symptoms and their association with subsequent physical activity using three approaches: 1) wrist accelerometry alone, 2) waist accelerometry alone, and 3) a combined approach using both measures. These models also examined between-subjects predictors including measures of overall functioning, mobility testing, and demographics. Level of fatigue reported in the previous interval was the strongest and most consistent predictor of subsequent lowered physical activity. Other significant predictors included age and functional mobility. Waist and wrist actigraphy estimates were modestly correlated with each other, yet multi-level models showed consistent results regardless of placement at wrist versus waist versus a combined waist/wrist approach. In conclusion, fatigue symptoms are a significant factor in predicting subsequent decreased physical activity in OA. Waist and wrist accelerometry methods produced different estimates of activity level in this population, but similar associations with arthritis symptoms.

